# Different bacterial and viral pathogens trigger distinct immune responses in a globally invasive ant

**DOI:** 10.1038/s41598-019-41843-5

**Published:** 2019-04-08

**Authors:** Philip J. Lester, Kaitlin H. Buick, James W. Baty, Antoine Felden, John Haywood

**Affiliations:** 1 Centre for Biodiversity and Restoration Ecology, School of Biological Sciences, Victoria University of Wellington, PO Box 600, Wellington, 6012 New Zealand; 2 Malaghan Institute of Medical Research, PO Box 7060, Wellington, 6242 New Zealand; 3 School of Mathematics and Statistics, Victoria University of Wellington, PO Box 600, Wellington, 6012 New Zealand

## Abstract

Invasive species populations periodically collapse from high to low abundance, sometimes even to extinction. Pathogens and the burden they place on invader immune systems have been hypothesised as a mechanism for these collapses. We examined the association of the bacterial pathogen (*Pseudomonas* spp.) and the viral community with immune gene expression in the globally invasive Argentine ant (*Linepithema humile* (Mayr)). RNA-seq analysis found evidence for 17 different viruses in Argentine ants from New Zealand, including three bacteriophages with one (*Pseudomonas phage PS-1*) likely to be attacking the bacterial host. Pathogen loads and prevalence varied immensely. Transcriptomic data showed that immune gene expression was consistent with respect to the viral classification of negative-sense, positive-sense and double-stranded RNA viruses. Genes that were the most strongly associated with the positive-sense RNA viruses such as the *Linepithema humile virus 1* (LHUV-1) and the *Deformed wing virus* (DWV) were peptide recognition proteins assigned to the Toll and Imd pathways. We then used principal components analysis and regression modelling to determine how RT-qPCR derived immune gene expression levels were associated with viral and bacterial loads. Argentine ants mounted a substantial immune response to both *Pseudomonas* and LHUV-1 infections, involving almost all immune pathways. Other viruses including DWV and the *Kashmir bee virus* appeared to have much less immunological influence. Different pathogens were associated with varying immunological responses, which we hypothesize to interact with and influence the invasion dynamics of this species.

## Introduction

Populations of invasive species may grow to dominate landscapes and environments, before collapsing to local extinction^1^. One of the classic examples of these boom-and-bust population dynamics is with the introduction of the common North American water-weed, *Elodea canadensis*, into Great Britain^2^. It was first observed in Scotland in 1842, but soon spread and grew to high densities in much of the UK and Ireland. Sections of the river Thames became impassable. Then a sudden decline occurred so that within 15 years the water-weed had completely disappeared from many regions and waterways^2^. Populations of invasive ants have also been known to display boom-and-bust dynamics^3^. Large populations that form great “rivers” of invasive ants can suddenly decline and disappear completely without human intervention. Pathogens and disease are frequently hypothesized as the cause of declines in invasive species populations, even in the absence of any direct evidence that they occur or influence their host population^1^.

Social insects such as ants were once believed to suffer few pathogens. The first virus infecting invasive ants was reported from red imported fire ants (*Solenopsis invicta*) only in 2004^4^. Since this time, five single-stranded RNA viruses and a single-stranded DNA virus have been found in this invasive ant^5,6^. These pathogens may decrease foraging performance, alter diet, and decrease the ants’ competitive ability in interspecific interactions^7,8^. Immune genes may also be up-regulated or down-regulated after infection with viruses or entomopathogens in fire ants^9,10^. Argentine ants (*Linepithema humile*) are another of the most widespread, abundant and damaging invasive ants^11^. Eleven viruses have been described from Argentine ants since their first description in 2015^12–14^. These viruses are from eight different viral subfamilies^12^ and likely have variable effects on their ant hosts. Three of the viruses are shared with honey bees (*Apis mellifera*): *Black queen cell virus* (BQCV), *Deformed wing virus* (DWV), and *Kashmir bee virus* (KBV). These “honey bee” viruses have also been observed to infect and replicate in other insects^15,16^, though Argentine ants appear to have higher viral loads when interacting with honey bees^14^. At any one time colonies of ants or bees are frequently co-infected with a range of viral and other pathogens referred to as the ‘pathosphere’^17–20^. Pathogens may interact to influence other pathogens within their hosts. In addition to the viruses, Argentine ants have a diverse bacterial community that spatially varies both internationally and even within countries like New Zealand^21^. Very little is known about the influence of these viruses, bacterial species, or the microbial pathosphere on Argentine ant physiology.

The immune response of any ant species to viral infection is poorly understood. Much more is known about how honey bees respond to viruses and other pathogens. McMenamin *et al*.^22^ reviewed honey bee and insect immune pathways. They described how the genome of honey bees encodes the major components of insect immune pathways, with a focus on genes influenced by viral infections. Gene expression patterns altered by viruses include those in the immune pathways: Jak/STAT (Janus kinase/Signal Transducer and Activator of Transcription), RNAi (RNA interference), Toll, Imd, and JNK (c-Jun N-terminal kinase). For example, viruses are known to increase expression of genes such as *hopscotch* (Jak/STAT pathway), *dicer* and *argonaute-2* (RNAi) in honey bees^23^. Infection of honey bees by different viruses from different viral families results in a pattern of immune gene expression with some common genes, but also in many distinct expression patterns^23^. The immune response to viral challenges in ants and bees also appears to be influenced by the presence of other pathogens, including bacteria^12,18,24^. Recent work in Argentine ants has indicated there may be a core set of immune genes that are influenced by infection with pathogens such as bacteria. These core immune genes include many in the Toll and Imd pathways^25^. Different pathogens could compete for host resources or may even be synergistic in their influence on the ant or bee physiology.

Our goal in this study was to examine the association of variation in viral community or pathosphere with immune gene expression in Argentine ants. We first used an RNA-seq approach to examine expression patterns of immune genes in Argentine ant communities with varying viral species and infection loads. We then focused on how four viruses are associated with the expression of four different immune pathways, represented by nine immune genes. Because the immune response to viruses may be influenced by other microbial pathogens, we also included in our analysis a putative bacterial pathogen (*Pseudomonas* spp.) that we previously identified as common in Argentine ants^21^. *Pseudomonas* bacteria are major pathogens in a wide range of both plants and animals^26,27^. Overall, we wanted to understand how different viruses are associated with the immune response in these globally invasive ants. Do all the viral pathogens have a similar influence on the ant’s immune physiology? How are different immune pathways within the ants influenced by different viruses and bacteria?

## Results

### Virus and immune gene analysis via RNA-seq

**Figure 1:**
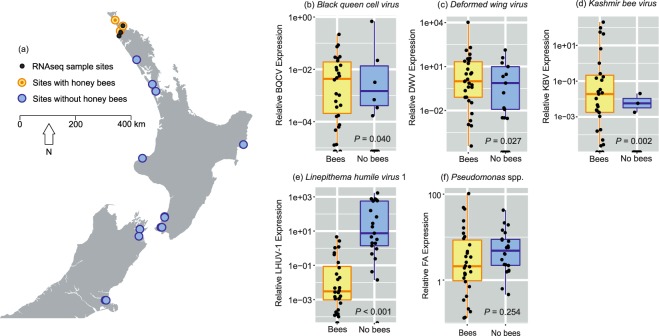
Sampling locations, virus and bacterial loads within Argentine ants. (**a**) Shows the geographic locations of the samples for the RNA-seq analysis, with 12 samples taken at three sites. The sites used for the RT-qPCR analysis encompassed the entire distribution of Argentine ants within New Zealand. (**b**–**e**) Show the results of RT-qPCR tests examining viral loads in ants when in the presence or absence of honey bees, and (**f**) shows infection levels from bacterial *Pseudomonas* spp. *P-*values are from permutational ANOVA.

We sampled ant populations for the RNA-seq analysis only from sites in Northland (Fig. 1a), so as to limit the influence of extraneous variables on the ant microbiota and gene expression. Seventeen viruses were putatively found in the RNA-seq analysis, including evidence for three different bacteriophage viruses. Six viruses were found in all samples analysed (i.e. *Linepithema humile partiti-like virus 1*, *Linepithema humile toti-like virus 1*, *Linepithema humile bunyan-like virus 1*, *Linepithema humile polycipivirus 2*, *Linepithema humile picorna-like virus 1* and *Linepithema humile virus 1;* Table 1), of which four had relatively consistent patterns of infection. Both *Linepithema humile partiti-like virus 1* and *Linepithema humile toti-like virus 1*, for example, showed a ≤ 10-fold level of variability in infection between samples. In contrast, the two viruses showing the most variability in viral abundance between RNA-seq samples were *Linepithema humile C-virus 1* with a 12,032-fold change between highest and lowest infection, and the *Deformed Wing Virus* with a 5,968-fold change between highest and lowest infection. Two other viruses that we assayed in the RT-qPCR analysis were also amongst the top five most variable virus loads between samples (*Linepithema humile virus 1* with 464-fold variation, and *Kashmir Bee Virus* with 62-fold variation). Our previous work has suggested that virus loads may change in ants when they are in the presence of honey bees^14^. The RNA-seq sampling did not provide sufficient data to examine this question statistically as, for example, only two of eight samples tested positive for KBV in sites without honey bees. The available data indicated that the range of infection loads was similar for many viruses in sites with and without honey bees. Some viruses, however, such as DWV and LHUV-1 had viral loads that were orders of magnitude higher when in the presence of honey bees (Table 1).

**Table 1:** Viruses and phages found in the RNA-seq data.

Viral species	Without honey bee hives			With honey bee hives			All sites combined		
	Mean	Range	*n*	Mean	Range	*n*	Mean	Range	*n*
*Linepithema humile partiti-like virus 1 (LhuPLV1)* ^†^	1.343	(0.295–2.739)	8	2.183	(1.784–3.008)	4	1.623	(0.295–3.008)	12
*Linepithema humile toti-like virus 1 (LhuTLV1)* ^†^	0.782	(0.224–1.434)	8	0.988	(0.500–1.635)	4	0.851	(0.224–1.635)	12
*Linepithema humile bunya-like virus 1 (LhuBLV1)* ^†^	0.978	(0.391–2.232)	8	0.333	(0.118–0.665)	4	0.763	(0.118–2.232)	12
*Linepithema humile polycipivirus 2 (LhuPcV2)*	0.518	(0.110–1.432)	8	1.187	(0.526–2.303)	4	0.741	(0.110–2.303)	12
*Linepithema humile picorna-like virus 1 (LhuPiLV1)*	0.067	(0.002–0.250)	8	1.688	(0.015–6.625)	4	0.607	(0.002–6.625)	12
*Linepithema humile virus 1 (LHUV-1)* ^†^	0.037	(0.007–0.065)	8	1.153	(0.162–3.081)	4	0.409	(0.007–3.081)	12
*Pseudomonas phage PS-1*	2.341	(0.427–6.802)	6	1.440	(0.379–2.371)	4	1.980	(0.379–6.802)	10
*Linepithema humile C-virus 1 (LhuCV1)* ^†^	2.449	(0.246–6.625)	4	2.330	(0.001–6.829)	3	2.398	(0.001–6.829)	7
*Deformed wing virus (DWV)* ^†^	0.001	(0.001–0.002)	3	0.948	(0.024–3.290)	4	0.542	(0.001–3.290)	7
*Kashmir bee virus (KBV)* ^†^	0.106	(0.069–0.142)	2	2.683	(0.265–4.248)	4	1.824	(0.069–4.248)	6
*Linepithema humile rhabdo-like virus 1 (LhuRLV1)* ^†^	8.875	(6.577–11.196)	3	1.503	(0.986–2.020)	2	5.926	(0.986–11.196)	5
*Rhopalosiphum padi virus*	1.711	(1.098–2.514)	3	1.075	(1.075–1.075)	1	1.552	(1.075–2.514)	4
*Shigella phage SfIV*	1.091	(0.859–1.271)	3	2.709	(2.709–2.709)	1	1.496	(0.859–2.709)	4
*Linepithema humile narna-like virus 1 (LhuNLV1)*	—	—	0	3.018	(1.114–5.868)	3	3.018	(1.114–5.868)	3
*Escherichia virus HK022*	1.982	(0.787–3.176)	2	0.540	(0.540–0.540)	1	1.501	(0.540–3.176)	3
*Salmonella phage SJ46*	3.317	(3.317–3.317)	1	—	—	0	3.317	(3.317–3.317)	1
*Thika virus*	3.317	(3.317–3.317)	1	—	—	0	3.317	(3.317–3.317)	1

For each virus, viral loads are expressed as the scaled sum of TMM-normalised TPM counts for all annotated transcripts present. TPM counts were also normalised by the respective Argentine ant library size. The rows are sorted firstly by the highest to lowest prevalence of the viruses in the 12 samples, and then by the average viral abundance. The average abundance estimates are only from sites where the virus occurs, i.e. values of 0 were excluded from the average virus estimates. Viruses marked with † were those confirmed via RT-PCR and Sanger sequencing.

**Figure 2:**
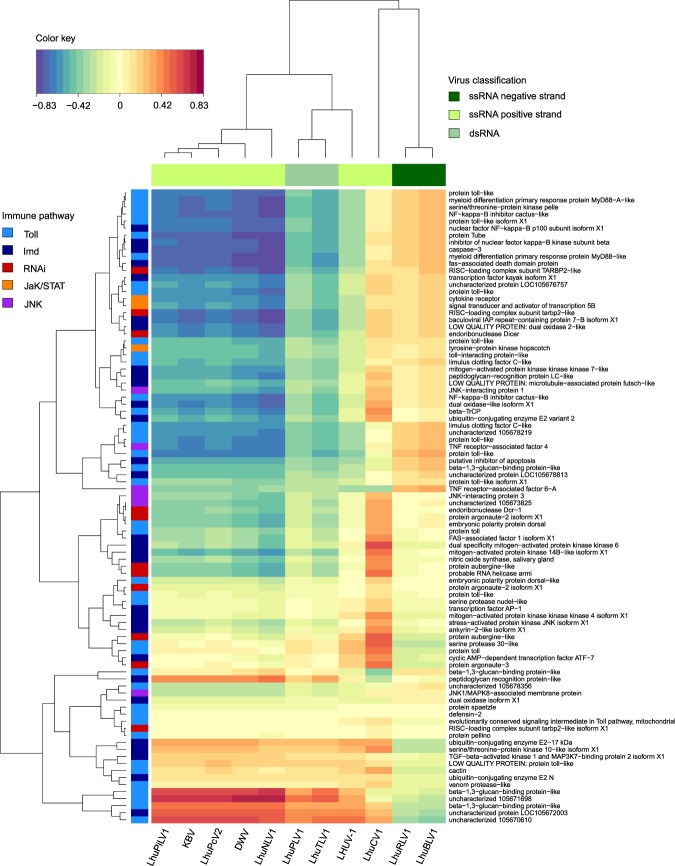
Heatmap showing the regression matrix between viral loads for 11 viruses and gene expression for 90 immune genes obtained after Partial Least Squares regression analysis. Virus types clustered together (top dendrogram, colour-coded in the top bar), suggesting that specific sets of immune genes are specifically associated with positive and negative single-stranded, and double stranded RNA viruses. We included immune genes associated with the Toll, Imd, RNAi, Jak/STAT and JNK immune pathways, colour-coded in the left-hand bar). The colour key from blue to yellow to red indicates the strength of the regression between each virus and immune gene.

The PLS-regression analysis aimed at finding associations between the detected viruses and known Argentine ant immune genes. Hierarchical clustering of viruses indicated that immune gene expression was generally consistent with respect to the viral classification of negative-sense, positive-sense and double-stranded RNA viruses (Fig. 2). The patterns of gene expression from infection were most distinct in association with infection by the two negative-sense RNA viruses LhuRV1 (*Linepithema humile rhabdo-like virus* 1) and LhuBLV1 (*Linepithema humile bunya-like virus* 1), while most positive-sense as well as dsRNA viruses appeared to elicit similar immune responses. LhuCV1 (*Linepithema humile C-virus* 1), a positive-sense RNA virus, did not show a comparable pattern to any other virus in terms of its association with immune gene expression. A large number of genes across all immune pathways were positively correlated with negative-sense RNA viruses. Interestingly, the same set of genes appeared down-regulated in response to dsRNA and most positive-sense viruses (see the top half of the heatmap). Surprisingly, genes that function within the RNAi and JaK/STAT pathways, which have been previously associated with anti-viral immune defence in bees^24^ were often negatively associated with positive-sense and dsRNA viruses, and appeared to be mostly activated by negative-sense viruses. In contrast, genes that were the most strongly associated with positive-sense RNA viruses were peptide recognition proteins assigned to the Toll and Imd pathways (*e.g*. beta-1,3-glucan-binding and peptidoglycan recognition proteins) and displayed negative associations with negative single-stranded RNA viruses.

### Virus, bacteria and immune gene analysis by RT-qPCR

**Figure 3:**
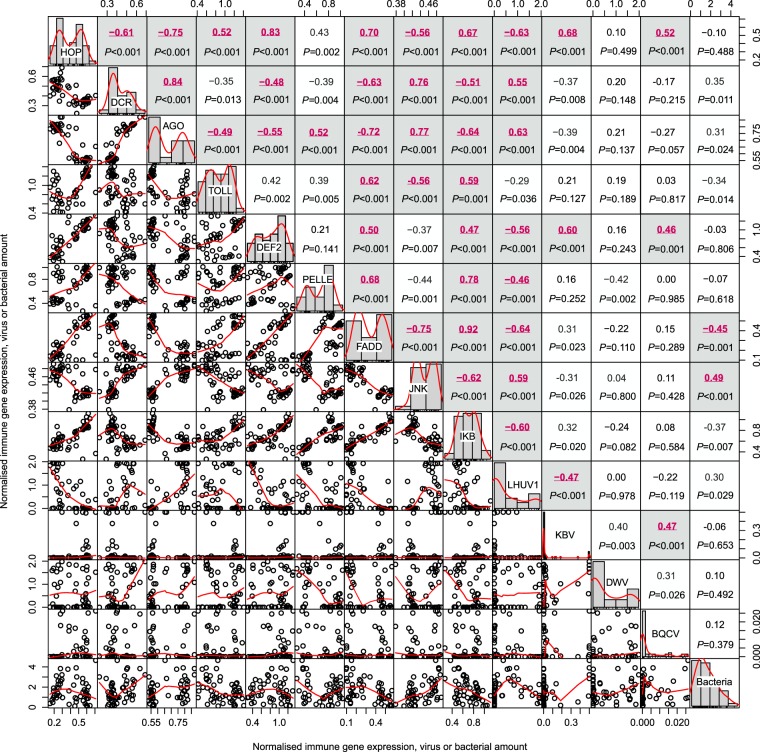
Spearman rank correlations and distribution plots between all immune genes, viruses and the *Pseudomonas* spp. bacteria quantified by RT-qPCR. Data were Box-Cox transformed prior to analysis. The immune genes were *tyrosine-protein kinase hopscotch* (HOP), *dicer-2* (DCR), *argonaute-2* (AGO), *protein toll* (TOLL), *defensin-2* (DEF2), *serine/threonine-protein kinase pelle* (PELLE), *fas-associated death domain protein* (FADD), *stress-activated protein kinase JNK* (JNK), and *inhibitor of nuclear factor kappa-B kinase subunit beta* (IKB). The viruses examined were *Linepithema humile virus* 1 (LHUV-1), *Kashmir bee virus* (KBV), *Deformed wing virus* (DWV), and *Black queen cell virus* (BQCV). The bacterial assay quantified *Pseudomonas* spp. Significant *P*-values (<0.05) are shown in grey boxes with red text and are after sequential Bonferroni adjustment.

Our RT-qPCR analysis examined viruses, bacteria and immune gene expression in samples of 30 ants sampled from around New Zealand. Of the viruses, the most prevalent to least prevalent were LHUV-1 (49 of 52 samples), followed by DWV (43/52), BQCV (32/52), and KBV (30/52). Comparison between species of viral infection loads is difficult due to PCR amplification constraints, though of all viruses LHUV-1 and DWV had an order of magnitude higher average level of infection than KBV and BQCV. BQCV appeared to have the lowest rate of infection or load within ants. Within viruses we observed a substantial amount of variation between samples. For DWV, there was a 276.3 million-fold increase in viral loads from lowest to most infected sample. Levels of variation were similarly high for LHUV-1 (a 16.7 million-fold change) and KBV (an 8.4 million-fold change). The bacteria *Pseudomonas* spp. was positive in all samples. It ranged in relative expression from 0.131–103.390 (a 788-fold level of variation). The comparison of sites with and without honeybees indicated significantly higher viral loads for BQCV, DWV and KBV in apiaries (Fig. 1b–d). In contrast, LHUV-1 viral loads in ants were orders of magnitude higher outside of apiaries, and there was no difference in *Pseudomonas* spp. loads (Fig. 1e,f). There were several significant correlations between viral infection loads. For example, LHUV-1 was negatively correlated with KBV (*r*_*s*_ = −0.47, *P* < 0.001, Fig. 3). BQCV was positively correlated with KBV (*r*_*s*_ = 0.47, *P* < 0.001, Fig. 3).

We observed much less variation in the expression of the immune genes. The nine genes showed a 3- to 12-fold level of variation between the minimum and maximum values. Spearman rank correlations indicated that the expression of any immune gene was typically highly correlated with other immune genes (Fig. 3). This result was expected for genes within pathways. For example, *dicer* and *Argonaut* are both within the RNAi pathway and their expression in ants was highly correlated (*r*_*s*_ = 0.84, *P* < 0.001, Fig. 3). Correlations between different immune genes were significantly positive and negative, likely indicating pathways have a mixture of activation of and inhibition of expression.

Our principal components analysis (PCA) showed that the five original, Box-Cox transformed (but unstandardized) pathogens were almost linearly independent: each linearly independent component had a single pathogen coefficient that explained the vast majority (≥93.1%; or ≥0.965^2^) of the component’s variation (Table 2). The ordering of the principal components from most to least variance gave an effective ordering of the variance in pathogen loads: the bacteria *Pseudomonas* spp., LHUV-1, DWV, KBV and BQCV. We then estimated a regression model for each immune gene on the five pathogen principal components (Table 3). These regression models suggest that the *Pseudomonas* spp. and LHUV-1 were important for the majority of the immune genes. Both DWV and KBV each significantly associated with only one of the nine immune genes. BQCV was not observed to be significantly associated with the expression of any immune gene. Note that the coefficients need *ceteris paribus* interpretations: the results for each immune gene are in response to a unit change in each pathogen principal component while holding all other variables constant, and should not be interpreted in isolation. The principal components are linearly independent by construction though, so there is no correlation between any of the predictors but the signs of the coefficients in each component are arbitrary (as in any PCA). Interestingly, however, the signs of the coefficients for the majority of the immune genes in *Pseudomonas* spp. were the opposite to those of LHUV-1 and that contrast is meaningful.

**Table 2:** Principal components analysis on the Box-Cox transformed (but unstandardized) viral and bacterial pathogens.

Factor	PC1	PC2	PC3	PC4	PC5
	*“Pseudomonas”*	“LHUV-1”	“DWV”	“KBV”	“BQCV”
*Pseudomonas* spp.	−0.970	−0.052	−0.235	0.039	0.001
LHUV-1	−0.033	0.994	−0.091	−0.041	−0.001
DWV	−0.235	0.084	0.965	0.083	0.002
KBV	−0.056	−0.036	0.075	−0.995	−0.001
BQCV	−0.001	−0.001	0.002	0.002	−1.000
Standard deviation	1.114	0.723	0.677	0.130	0.008
Proportion of variance	0.554	0.233	0.205	0.008	<0.001
Cumulative proportion	0.554	0.787	0.992	1.000	1.000

The analysis produced five independent components, each with a single coefficient that explained the vast majority (≥93.1%; or ≥0.965^2^) of the component variation. Hence each component was strongly associated with a single pathogen that is named in quotation marks. The five putative pathogens were *Pseudomonas* spp., *Linepithema humile virus 1* (LHUV-1), *Deformed wing virus* (DWV), *Kashmir bee virus* (KBV), and Black queen cell virus (BQCV).

**Table 3:** Linear regression results showing the estimated coefficients (standard errors) of the relationships between the immune genes and the five principal components, shown here as the five pathogens from the analysis presented in Table 2.

Pathway & gene	PC1	PC2	PC3	PC4	PC5	*r* ^2^
	*“Pseudomonas”*	“LHUV-1”	“DWV”	“KBV”	“BQCV”	
**Jak/STAT pathway**						
*Hopscotch* (Hop)	0.011 (0.015)	−0.147 (0.024)***	0.022 (0.025)	−0.321 (0.133)*	−3.724 (2.253)	0.509
**RNAi pathway**						
*Dicer* (DCR)	−0.038 (0.010)***	0.064 (0.016)***	0.023 (0.017)	0.160 (0.087)	0.569 (1.469)	0.443
*Argonaute-2* (AGO)	−0.044 (0.010)***	0.103 (0.016)***	0.006 (0.017)	0.113 (0.090)	1.263 (1.523)	0.569
**Toll pathway**						
*Toll* (Toll)	0.094 (0.035)**	−0.051 (0.053)	0.081 (0.057)	0.146 (0.297)	4.975 (5.045)	0.199
*Pelle* (Pelle)	0.062 (0.021)**	−0.188 (0.032)***	−0.136 (0.034)***	0.182 (0.180)	1.020 (3.052)	0.564
*Defensin-*2 (DEF2)	−0.021 (0.030)	−0.279 (0.046)***	0.061 (0.049)	−0.357 (0.256)	−3.309 (4.353)	0.473
**Imd pathway**						
*FADD* (FADD)	0.075 (0.013)***	−0.131 (0.021)***	−0.010 (0.022)	−0.009 (0.114)	−0.649 (1.943)	0.612
*JNK* (JNK)	−0.014 (0.003)***	0.021 (0.004)***	−0.003 (0.004)	0.001 (0.022)	0.037 (0.380)	0.564
*IKB* (IKB)	0.085 (0.018)***	−0.142 (0.027)***	−0.033 (0.029)	0.062 (0.150)	1.922 (2.552)	0.535

The immune genes are arranged by immune pathways. The r^2^ values are for the overall fit of each model. *p-value < 0.05*, <0.01**, <0.001***. ^†^The intercept coefficients on all models are not shown, but were all statistically significant (*p* < 0.001) and ranged between 0.413–0.935.

### Single ant analysis of viruses and immune genes by RT-qPCR

In order to further understand viral loads and the Argentine ant immune system, we also examined virus and immune gene expression in individual Argentine ants. All ants were collected from the same site, colony and time. The viruses showed an extremely high level of variation, with a 160-fold level of variation for LHUV-1 and 30-fold for DWV (Table 4). In contrast the immune genes *defensin-*2 (DEF2), *tyrosine-protein kinase hopscotch* (HOP), and *argonaute-2* (AGO) showed relatively lower levels of variation in expression between ants.

**Table 4:** An analysis of patterns of viral load and immune gene expression in single ants, all of which were sampled from the same colony at the same time.

Ant	Viruses		Immune genes		
	LHUV-1	DWV	DEF2	HOP	AGO
1	0.078	0.000	1.185	2.163	1.456
2	0.795	0.000	0.697	1.868	0.891
3	1.748	0.000	0.431	2.527	1.655
4	7.340	0.836	0.640	1.916	1.593
5	12.471	0.163	0.813	0.773	2.579
6	3.476	0.353	0.827	0.677	4.083
7	2.250	1.824	0.985	0.699	3.078
8	0.208	0.211	0.422	0.575	3.244
9	0.765	0.066	0.630	0.707	3.329
Range: minimum	0.078	0.066	0.422	0.575	0.891
Range: maximum	12.471	1.824	1.185	2.527	4.083
Fold change	160.6	27.8	2.8	4.4	4.6
Coefficient of variation	1.277	1.570	0.336	0.589	0.442

Virus loads were extremely variable between ants, relative to the variation observed in immune gene expression. The viruses were *Linepithema humile virus 1* (LHUV-1) and the *Deformed wing virus* (DWV). The immune genes examined were *defensin-*2 (DEF2), *tyrosine-protein kinase hopscotch* (HOP), and *argonaute-2* (AGO).

## Discussion

We found that invasive Argentine ants in New Zealand have a diverse and highly variable pathosphere. Viral infections varied between sites by several orders of magnitude and were associated with a variable immune response. Of the viruses examined in detail via RT-qPCR, the *Deformed wing virus* (DWV) and the *Linepithema humile virus 1* (LHUV-1) were highly variable with some ants showing a 276 million-fold difference in load between sites. The immune response appeared to be greatest to the bacteria *Pseudomonas* spp. and LHUV-1 infections, while much reduced for BQCV.

The field of eco-immunity is relatively new and still developing. Cornet *et al*.^28^ highlight how host immunity is at the core of major theories for invasion biology. Changes in pathogen communities can result in a reallocation of resources from immune defences to beneficial traits associated with increased reproductive output or invasive potential. The potential for such a reallocation makes sense if different pathogens and pathogen communities invoke varying costs for their hosts^29^. The presence of honey bees appeared to be associated with a substantial alteration of the viral community within ants (although we acknowledge it is difficult to be conclusive in this result as ant-bee interactions were only observed in northern sites). We observed variation in infection and immune responses throughout the sampling sites. In other ants, different viral pathogens have previously been demonstrated to incur different phenotypic effects. For example, SINV-1 significantly reduced fire ant (*Solenopsis invicta*) queen weight but not reproductive output, while SINV-2 reduced reproductive output but not weight^10^. Each *Solenopsis invicta* virus appears to induce a different immune response in their ant host^10^. Transcriptomic data analysis indicates that different viruses similarly appeared to induce a different response in Argentine ants, with responses largely grouped by viral classification. Interestingly, PCA analysis indicates that different viruses of similar types appeared to be associated with different immune responses. This observed variation in immune response to different viruses might be induced by a range of different mechanisms, which include some virus families having molecular machinery that inhibits certain antiviral immune responses^30^.

Microbial species including gut microbiota have been hypothesized to influence immune responses, pathogen communities, and ultimately their host’s invasion success^28^. Perhaps the most extreme example of bacteria influencing viral infections in insects is the intracellular bacteria *Wolbachia* inhibiting infection of mosquitoes from the virus causing dengue fever^31^. An extreme example of viruses influencing bacterial infections are phages, which have even been described as mutualists for the hosts of pathogenic bacteria^32^. Our study provides the first evidence of phages in Argentine ants. *Pseudomonas* is a Gram-negative bacterial pathogen with a wide host range that includes plants, insects and animals^26,27^. It can be a virulent bacterial pathogen of insects with an LD_50_ of less than 10 bacterial cells^26^. *Pseudomonas* has been found in Argentine ants^21,33,34^, although it is yet to be confirmed as an ant pathogen. Gram-negative bacteria have been associated with an increased immune response along the Imd pathway^24^, which we observed, though this bacterium also appeared to influence most of the immune genes that we quantified. Interestingly, the sign of the coefficients for the response of the immune genes to *Pseudomonas* was typically the opposite for that to LHUV-1 (Table 3). Pathogen loads within ant samples were positively correlated for some species, and negatively correlated for others. Different microbial pathogens are clearly associated with different immune responses, which may be related to variation in replication strategies and virulence among different classes of virus, or among different viruses. For instance, in mosquitoes, the Toll pathway is efficient against *Flavivirus*, but not *Alphavirus*, both positive single-stranded RNA viruses^35^. Given that different virus species within the same class can illicit such different immune responses, it is easy to conceive that different sets of genes will be affected by different viral classes. Viljakainen *et al*.^25^ similarly found viruses and bacteria to jointly influence the immune response of Argentine ants, via both up- and down-regulation of different genes. Overall, it appears the model of a ‘pathosphere’^18–20^ seems appropriate here for Argentine ants. The pathogen community likely interacts and jointly influences the host’s immune response, with the phages perhaps influencing some bacteria. While our models explained a high proportion of the variation in immune gene expression, other microbial taxa undoubtedly influenced the health and immune response of these ants. Argentine ants have a diverse bacterial community^21^ and other viruses^12^ that we did not quantify, or that are still unknown to science.

Currently little is known about the influence of these viruses on ants. For the viruses and bacteria that we analysed, our results suggest that these pathogens are associated with a strong immune response, especially LHUV-1 and *Pseudomonas* spp. A comparatively much-reduced response was observed in association with DWV, KBV and BQCV infection levels. Samples of ants showed high levels of variation in the viral loads. For example, in the composite samples of 30 ants in the RT-qPCR analysis, there was a 16.7 million-fold increase in LHUV-1 loads from lowest (non-zero) to most infected sample. The overall levels of variation are consistent with variation in viral loads observed in honey bees^36,37^, bumble bees^36^, and wasps^38^. Our analysis of within-site variation for single ants showed a 28-fold variation for DWV, and a 161-fold difference between the highest and lowest level of LHUV-1 infection. These levels of variation between ants are of some concern for sampling and attempts to understand the influence of viruses on ant populations. If high viral titres are only observed in a small percentage of ants within a population then a single ant with, for example, a ~200-fold higher viral load in some individuals will skew estimates of viral loads.

Variation in observed immune gene expression was much less than for viruses. The samples of 30 pooled ants showed immune genes varied by 3- to 12-fold. For single ants, only a 3- to 5-fold level of variation was observed for the three genes we examined (*defensin-*2, *tyrosine-protein kinase hopscotch*, and *argonaute-2*). It is noteworthy that the patterns of expression for nearly all the immune genes were correlated both within and between immune pathways. We expected that *dicer* and *argonaute* expression would be highly correlated because they are within the RNAi pathway. Surprisingly, nearly all the other genes associated with the other immune pathways examined were also significantly and highly correlated with *dicer* and *argonaute* (Fig. 3). Some genes were positively and some negatively correlated, as has previously been observed in pathogen infection in Argentine and other ants^10,25^. The inter-relatedness of the immune system response, bacterial and viral infections supports an eco-immunological framework^28,39^ and the hypothesis that the microbial community within Argentine ants can exert an immunological cost. The extent of that immunological cost in relation to variable pathogen pressure, and whether or not it can induce population collapses of this invader^40^, or promote variation in its ecological success, remains to be determined.

## Methods

### Ant collection and RNA extraction

Ants were collected using an aspirator from eighteen locations across New Zealand (Fig. 1a). In locations to the north of New Zealand, Argentine ants were collected from honey bee (*Apis mellifera*) apiary sites where they were observed infesting hives and attacking bees. All ants were collected alive, immediately snap frozen in liquid nitrogen, and then stored at −70 °C until RNA extraction. RNA for the RNA-seq analysis was extracted using Direct-Zol RNA Microprep kit for the RNA-seq experiment, and Direct-Zol RNA Miniprep for the RT-qPCR experiment (Zymo Research, CA, USA). Briefly, ants were homogenized in 300, 600 and 100 µL of TRIzol Reagent (Life Technologies/ThermoFisher Scientific) for the Microprep, Miniprep for samples of pooled ants and Miniprep for single ant extractions, respectively. Pooled samples consisted of 30 ants from single sites. Homogenates were clarified by centrifugation and RNA purified following manufacturer’s instructions. For the RNA-seq samples, final RNA concentrations and integrity were determined using a BioAnalyzer (Agilent technologies, USA) and the RNA stored at −70 °C until samples were selected and shipped for sequencing. For the RT-qPCR samples, we determined RNA concentrations using a NanoDrop One (ThermoFisher Scientific) and likewise stored the samples at −70 °C until processed.

### Virus and immune gene analysis via RNA-seq

Twelve samples were selected for RNA sequencing, based on a preliminary RT-qPCR analysis of virus infection using RNA extracted from 30 ants per sample. We included samples collected in sites with and without honeybee hives, and selected samples that spanned a wide range of viral loads. RNA samples were stored and shipped in RNAStable (Biomatrica, USA) for sequencing by Annoroad (China). Libraries were prepared using the NEBNext Ultra RNA library Prep Kit for Illumina according to the manufacturer’s instructions and 150 bp paired-end reads were sequenced on a single lane using Illumina HiSeq platform. Data pre-processing included the removal of read pairs when either one read contained adapter contamination of more than 5 nucleotides, contained more than 10% of ambiguous nucleotide calls or contained more than 50% of low-quality base calls (Phred score <20, *i.e*. 99% accuracy).

Clean paired-end reads were aligned to the Argentine ant reference genome^41^ with HISAT 2.1.0 with default parameters^42^ to produce sample-specific BAM files. We then fed the BAM output into StringTie 1.3.4^43^ to generate GTF files, using the -e argument to restrict the assembly to transcripts matching the Argentine ant *RefSeq* genome records (GCF_000217595.1). We generated a raw transcript counts matrix at the gene level using the authors’ *prepDE.py* script. We imported the raw counts matrix into R as a *DGEList* object using the *edgeR* 3.22.3 package^44^, together with one of the GTF files created by *StringTie* as a source of information on each gene. In order to filter the raw counts matrix for low expressed transcripts we first computed counts per million (CPM), then discarded transcripts that were not expressed in at least 3 samples at more than 1 CPM. We then applied a TMM normalisation on the filtered raw count data. Expression data in CPM for 90 annotated genes likely associated with immune pathways were extracted and transformed into FPKM using transcript lengths, and inputted as a matrix for PLS-regression analysis. We selected the immune genes based on the KEGG Pathway database as well as searching the Argentine ant *RefSeq* genome records for genes known to be associated with immunity in closely related organisms. Of all 90 genes included in the analysis, 40 (44%) were in the Toll immune pathway, 30 (33%) were in the Imd pathway, 11 (12%) in the RNAi pathway, 6 (7%) in the JNK pathway, and 3 (3%) in the Jak/STAT pathway.

Reads that did not align to the Argentine ant genome in the *HISAT* step were *de novo* assembled using *Trinity* 2.3.2 with default parameters. We quantified assembled transcript expression within *Trinity* using *eXpress* 1.5.1 (Roberts and Pachter 2012), yielding a TMM-normalised TPM matrix at the gene level. We then aligned the assembled transcripts to various reference databases in order to screen for and quantify viral transcripts: we used BLAST 2.2.3^45^ to run BLASTx searches of the NCBI viral protein database as well as of a more recently published Argentine ant novel viruses database^12^, using a *e-value* cut-off of 10^−5^. We discarded hits that were less than 100 bp long and returned less than 90% identity with the target sequence. From the filtered BLASTx output, we selected a single best hit per assembled transcript based on the highest bitscore. In order to compute viral loads, we first summed TPM values for all genes belonging to each identified virus. Then, we used the Argentine ant library size to normalise viral loads to host tissue among samples. Log-transformed TMM-normalised transcripts per million (TPM), normalised to Argentine ant read counts, were stored as a matrix for later analysis.

In order to explore associations between Argentine ant immune response and viral pressure, we used the immune gene expression and viral load matrices as input for a Partial Least Square analysis (PLS) in regression mode with eight components, using *mixOmics*^46^. We plotted a heatmap of similarity matrix showing the association between viral loads and immune gene expression, based on the two components that were significant. All remaining statistical analyses were performed in R 3.5.1^47^.

### Virus, bacteria and immune gene analysis by RT-qPCR

cDNA was generated from 500 ng RNA/sample using qScript XLT cDNA SuperMix (QuantaBio, MA, USA) in 20 µL reactions. The cDNA was diluted to a final volume of 212 µL with water and 8 µL/sample loaded into each well of the MicroAmp Fast Optical 96-Well Reaction Plate (Applied Biosystems/ThermoFisher Scientific) for RT-qPCR. Each reaction also contained: 10 µL PowerUp SYBR Green Master Mix (Applied Biosystems/ThermoFisher Scientific), and forward and reverse primers at final concentrations of 300 nM. Plates were analysed on a QuantStudio 7 Flex Real-Time PCR System (Applied Biosystems) using fast PCR cycling conditions (50 °C, 2 min; 95 °C, 2 min; 40 cycles of 95 °C, 1 s, 60 °C, 30 s). Cycle threshold (*Ct*) values were used to calculate target (*Ct*_target_) levels relative to the reference (*Ct*_reference_) genes using the equation (2^(−*Ct*_target_))/(2^(−*Ct*_reference_)). We used the R package *lmPerm* (permutation ANOVA for linear models; Wheeler & Torchiano^48^) to examine for differences in relative virus loads between populations of Argentine ants collected near bee hives and from areas without hives. The targets of the primers used for the above analysis, presence, identity, and, for selected species, viral replication, were confirmed via Sanger sequencing.

*Performance Analytics*^49^ was used within the R statistical environment to plot the data, following Box-Cox transformations on each variable in the *car* package^50^ to help reduce the influence of extreme values. Spearman correlation coefficients were calculated between immune gene expression and viral/bacterial loads, with a sequential Bonferroni adjustment for the *p-values*. We used principal components analysis to represent the unscaled variation in the five Box-Cox transformed pathogen loads as five linearly independent (latent) components, then linear regression models were used to relate the response of each immune gene to the five independent pathogen components.

## Data Availability

Supplementary information, Scripts and data can be downloaded at https://zenodo.org/record/2609697#.XJp7KBMvNTY under 10.5281/zenodo.2609697; RNA-seq reads can be accessed on the NCBI SRA repository (BioProject ID PRJNA528845, http://www.ncbi.nlm.nih.gov/bioproject/528845).
